# Sperm Binding to Oviduct Epithelial Cells Enhances TGFB1 and IL10 Expressions in Epithelial Cells as Well as Neutrophils *In Vitro*: Prostaglandin E_2_ As a Main Regulator of Anti-Inflammatory Response in the Bovine Oviduct

**DOI:** 10.1371/journal.pone.0162309

**Published:** 2016-09-23

**Authors:** Mohamed Samy Yousef, Mohamed Ali Marey, Nina Hambruch, Hiroyuki Hayakawa, Takashi Shimizu, Hassan Ali Hussien, Abdel-Razek Khalifa Abdel-Razek, Christiane Pfarrer, Akio Miyamoto

**Affiliations:** 1 Graduate School of Animal and Food Hygiene, Obihiro University of Agriculture and Veterinary Medicine, Obihiro, 080–8555, Japan; 2 Department of Theriogenology, Faculty of Veterinary Medicine, Assiut University, Assiut, Egypt; 3 Department of Theriogenology, Faculty of Veterinary Medicine, Damanhur University, Behera, Egypt; 4 Department of Anatomy, University of Veterinary Medicine Hannover, Hannover, D-30173, Germany; 5 Genetics Hokkaido Association, Shimizu-cho, 089–0103, Japan; University of Bonn, GERMANY

## Abstract

Sperm are allogenic to the female genital tract; however, oviducts provide optimal conditions for survival and capacitation of these non-self cells until fertilization. Recently, we showed that oviduct-conditioned media and prostaglandin E_2_ (PGE_2_) suppress sperm phagocytosis by polymorphonuclear neutrophils (PMNs) under physiological conditions. We hypothesized that sperm binding to bovine oviduct epithelial cells (BOECs) could change the local innate immunity via PGE_2_. As the first step to obtain basic information, sub-confluent BOEC monolayers were co-cultured with swim-up sperm for 2 h. BOECs with viable bound sperm were cultured for an additional 3, 6, 12, or 24 h. Then, we confirmed the impact of the sperm-BOEC binding on both BOECs and PMN gene expression. Immunohistochemistry revealed that BOECs strongly express TGFB1 and IL10 in the oviduct. Sperm binding to BOECs in culture induced the anti-inflammatory cytokines (*TGFB1* and *IL10*) and PGE_2_ production by BOECs. Exogenous PGE_2_ *in vitro* suppressed pro-inflammatory cytokine expression (*TNF* and *IL1B*) in BOECs. Moreover, pre-exposure of PMNs to BOEC-conditioned media suppressed the *TNF* expression, but the BOEC media co-cultured with sperm stimulated PMNs to express *TGFB1* and *IL10*, with increasing PGE_2_ secretion. Of note, exogenous PGE_2_ led PMNs *in vitro* to decrease their *TNF* expression and increase anti-inflammatory cytokines expression. Our findings strongly suggest that BOECs provide an anti-inflammatory environment under physiological conditions and the sperm-BOEC binding further strengthens this milieu thus suppresses PMNs in the bovine oviduct. PGE_2_ is likely to drive this stable anti-inflammatory environment in the oviduct.

## Introduction

It is widely accepted that oviduct is not only a vehicle for gametes transportation and fertilization, but it also functionally manages, responds, and supports sperm to ensure successful fertilization [1]. Moreover, oviduct provides a suitable environment for early embryonic development. Co-incubation with oviductal epithelial cells provides a suitable environment for sperm capacitation [2–6]. Additionally, the oviductal fluid at the pre-ovulatory phase is highly effective in maintaining the viability and motility of bovine sperm [7].

Sperm are foreign organisms to the female reproductive tract; therefore, in theory, a hostile reaction from the maternal immune system against the sperm could be expected. However, sperm are protected and stored in the oviduct. The mammalian oviduct is considered as a refuge for sperm, and it does not respond to insemination with an influx of leukocytes like the vagina, cervix, and uterus do [8]. Nonetheless, different immune cells, including polymorphonuclear neutrophils (PMNs), were found in the oviductal fluid of ewes and cows under physiological conditions [9,10]. Cytokines are key signals of the mucosa-associated immune system necessary for normal immunological homeostasis [11]. We have previously shown that bovine oviduct epithelial cells (BOECs) drive a variety of cytokines [12], thereby regulating local innate immune functions. It is well known that pro-inflammatory cytokines (e.g. *TNF* and *IL1B*) have a detrimental effect on sperm [13–15], whereas, anti-inflammatory cytokines (e.g. *TGFB1* and *IL10*) can create an immunosuppressive state in the mucosal environment [16]. This environment could partly account for sperm survival in the oviduct.

Interactions between sperm and oviductal epithelial cells induce expression of novel genes, resulting in changes of the biochemical environment in mice [17], pigs [18], and cows [19]. BOECs produce prostaglandin E_2_ (PGE_2_) and prostaglandin F_2α_ (PGF_2α_), which are actively involved in the regulation of oviductal contractions [20,21]. Additionally, binding of viable sperm to BOECs *in vitro* increases the local production of PGE_2_ [22]; however, not much is known about the effects of PGE_2_ in the local immunological environment of the oviduct. The sperm-BOEC binding model has often been used to examine the interactions between sperm and oviductal cells, and to explain how the oviduct selects and protects sperm. In addition, PMNs were found in the bovine oviductal flush [10], which may be affected by sperm-BOECs binding during sperm storage in the oviduct. As the first step to obtain basic information, in the present study, we used this *in vitro* model to investigate the expression and production of certain anti-inflammatory cytokines (TGFB1, IL10 and PGE_2_) and the expression of pro-inflammatory cytokines (IL1B and TNF), in response to the sperm-BOEC_S_ binding which might modulate the local immunological environment to steer it away from the maternal immune attack in the oviduct. The investigations were extended to determine the immunological impact of sperm-BOEC binding on the above gene expressions of PMN in culture.

## Materials and Methods

### Ethics Statement

Animal experiments described in this article were conducted in accordance with the Guiding Principles for the Care and Use of Research Animals Promulgated by Obihiro University of Agriculture and Veterinary Medicine, Japan. The protocol was approved by the Committee on the Ethics of Animal Experiments of the Obihiro University of Agriculture and Veterinary Medicine (Permit number 25–101).

### Isolation and culture of BOECs

Female reproductive tracts were opened and macroscopically examined (to be free of inflammation, pus, and abnormal color) in a local slaughterhouse (Hokkaido Livestock Co., Doto Plant Tokachi Factory; 1–1, Kita 2-chome, Nishi 24-jo, Obihiro, Hokkaido, Japan). The phase of the estrous cycle was identified as previously reported [23], based on the appearance, weight, and colour of the corpus luteum, and the follicular diameter. Only healthy oviducts in the pre-ovulatory phase were selected. Oviducts were ligated from both ends, immersed in phosphate buffer saline without calcium or magnesium (PBS^-/-^) supplemented with 0.3% gentamicin (Sigma-Aldrich, Steinheim, Germany) and amphotericin B (Sigma-Aldrich, Steinheim, Germany), and then transported to the laboratory. In the laboratory, oviducts were separated from the surrounding connective tissue, rapidly rinsed in 70% ethanol for disinfection, and rinsed three times with PBS^-/-^. Epithelial cells were isolated and cultured as previously described [24] with minor modifications. Briefly, BOECs from three to four cows were mechanically dislodged by gentle squeezing in a stripping motion with forceps. The cells were then pooled, purified, and cultured in DMEM/F12 (Dulbecco's Modified Eagle Medium: Nutrient Mixture F-12, Gibco, Grand Island, USA) supplemented with 2.2% NaHCO_3_, 0.1% gentamicin, 1% amphotericin, and 10% heat-inactivated fetal calf serum (FCS) (BioWhittaker, Walkersville, MD, USA) in 6-well culture dishes (Nalge Nunc International, Roskilde, Denmark) for 4 or 5 days at 38.5°C in 5% CO_2_ and 95% air until a monolayer was formed. The cells were trypsinized (0.05% trypsin EDTA; Amresco, Solon, OH, USA), re-plated in 12-well culture dishes at a density of 3×10^4^ cells/mL, and cultured until sub-confluence. The BOEC monolayer from the second passage was primed with 100 pg/mL estrogen (E_2_) (Sigma-Aldrich, Steinheim, Germany) and 1 ng/mL (P_4_) (Sigma-Aldrich, Steinheim, Germany). The concentration of these steroid hormones was maintained at levels similar to their physiological levels in the bovine oviduct during the pre-ovulatory period *in situ* [23]. The purity of BOECs was confirmed by reacting the cultured cells with monoclonal antibodies against cytokeratin (anti-cytokeratin, CK1) to then perform immunostaining. The cells in culture showed a characteristic epithelial morphology. Approximately 98% of the cells stained positively for CK1 antibodies.

### Sperm preparation

Frozen 0.5 mL semen straws were obtained from three highly fertile Holstein bulls from the Genetics Hokkaido Association (Hokkaido, Japan). Nine straws (three straws from each bull) were thawed in a water bath at 38°C for 30 sec, pooled, and washed 3 times using a modified Tyrode’s albumin, lactate, and pyruvate medium (Sp-TALP; 6.66g/L NaCl, 0.24g/L KCl, 0.168g/L NaHCO_3_, 0.062g/L NaH_2_PO_4_-2H_2_O, 0.102g/L MgCl_2_-6H_2_O, 0.9g/L D-Glucose, 1.12g/L Na lactate, 0.294g/L CaCl_2_, 2.383g/L HEPES in Milli-Q water). Sperm were then separated using a swim-up procedure described by [25]. Briefly, four 0.25 mL aliquots of sperm suspension were layered under l mL aliquots of Sp-TALP medium in 15 mL Falcon tubes. After 1 h of incubation at 39°C, the top 0.85 mL from each tube was aspirated, pooled and centrifuged at 1000 *g* for 10 min. The pellet was washed by centrifugation in fresh Sp-TALP, and the final pellet was reconstituted in 0.5 ml of Sp-TALP for the future use. The progressive motility of the recovered sperm was assessed by visual examination under a light microscope equipped with a stage warmer. Acrosome integrity was detected through a dual staining procedure with a Trypan Blue supravital stain and a Giemsa stain, as described by [26].These swim-up sperm were used in subsequent experiments.

### Isolation and preparation of PMNs

PMNs were isolated as previously described [10] with minor modifications. Blood collections were conducted at the Field Center of Animal Science and Agriculture of Obihiro University, and all experimental procedures complied with the guidelines for the care and use of agricultural animals at Obihiro University. Heparinized blood from a multiparous Holstein cow during luteal stage was collected and mixed with equal volume of PBS^-/-^, slowly layered over Ficoll-paque solution (Lymphoprep, Axis Shield, Oslo, Norway), and centrifuged at 1000 ×g for 30 min at 10 ^o^C. PMNs layer was mixed with Ammonium chloride lysis buffer (NH_4_Cl 155mM, KHCO_3_ 3.4 mM, EDTA 96.7μM) for 10 seconds, then centrifuged at 500 ×g for 10 min at 10°C in order to purify PMNs from red blood cells. After centrifugation, the cell pellet was washed twice by PBS^-/-^. The purity of PMNs as evaluated by flow cytometry was > 98% and the viability was around 99% as assessed by Trypan blue staining.

### Co-culture of sperm with BOECs

**Fig 1:**
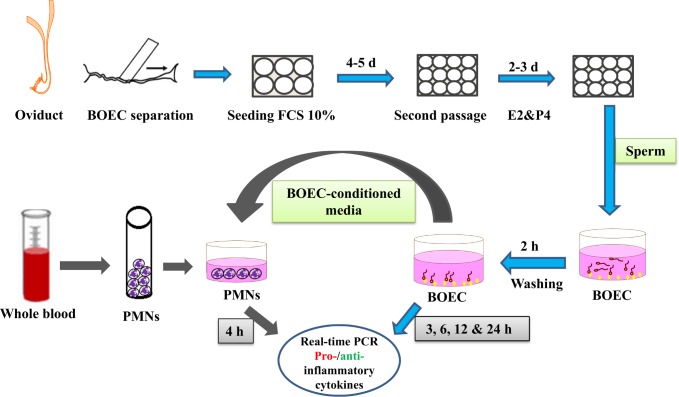
Flow diagram of the main experimental design. BOEC (bovine oviduct epithelial cells) monolayers co-cultured with sperm at different time points (3, 6, 12 and 24 h)**.** PMNs (polymorphonuclear neutrophils) isolated from whole blood and co-cultured for 4 h with BOEC-conditioned media. The cells were collected to analyse immune-related gene expressions using real-time PCR.

Sub-confluent BOEC monolayers in the second passage were pre-incubated for 1 h in co-culture medium (DMEM/F12; HEPES modification; 0.85 g/L NaHCO_3_, 50 mg/L gentamicin, and 56 mg/L ascorbic acid; Sigma-Aldrich, Steinheim, Germany) supplemented with 0.1% BSA and sodium selenite (5 mg/L). After pre-incubation, BOEC monolayers were co-cultured with the swim-up sperm (2 × 10^5^ sperm/mL) for 2 h to ensure the binding between sperm and BOECs. The supernatant containing dead and unattached sperm was removed and rapidly replaced by a pre-warmed co-culture media and incubated for 3, 6, 12, and 24 h at 38.5°C in 5% CO_2_ (Fig 1). Sperm motility and binding to epithelial cells was observed under a light microscope with stage warmer. Sperm bound to BOECs (about 50% from the initial number) showed high motility by more than 90%; the detached sperm were very few during the experimental period (24 h). A BOEC monolayer without sperm served as the control. At each time point, the medium was removed and cells were trypsinized. Then, cells were washed twice with PBS^–/–^and resuspended in 300 μL PBS^–/–^. Cell viability was estimated using Trypan blue staining. Using this technique, we confirmed that at each time point of plating as well as at the end of the experiment, plates had more than 95% viability. The remaining cells were again separated by centrifugation at 300 *g* for 10 min at 4°C, lysed with Trizol (Invitrogen, Carlsbad, USA), and stored at -80°C until RNA extraction. Conditioned media were collected from BOEC cultures after 12 and 24 h, centrifuged at 1000 *g* to remove the sperm, and the supernatants were stored at -80°C for further use and analyses (*n* = 5 for each time point).

Prior to determining the effect of sperm-BOEC binding on the gene expression of BOECs, the expression of our interested cytokines (*TGFB1*, *IL10*, *TNF*, *IL1B* and *PTGES*) were measured in sperm, BOEC and sperm + BOEC. The data showed very low mRNA expression in sperm (less than 5%) compared to those of BOEC or sperm + BOEC. Thus, our experiment was directed to determine the differences of mRNA expressions between BOECs (as a control) and sperm-BOEC at different time points.

### Effect of exogenous PGE_2_ in the gene expressions of TGFB1 and IL10 (anti-inflammatory cytokines), TNF and IL1B (pro-inflammatory cytokines) and PTGES in BOECs

The sub-confluent BOEC monolayers (second passage) were washed twice and cultured in medium supplemented with 0.1% FCS in combination with 3 different concentrations of PGE_2_ (3.5, 35 and 350 ng/mL that equivalent to 10^−8^, 10^−7^ and 10^−6^ M; Sigma-Aldrich, Steinheim, Germany) and then incubated for 24 h. This concentration range was selected based on the PGE_2_ levels found in oviduct flushes during ovulatory periods [10] and the PGE_2_ levels measured in the BOEC-conditioned media with or without the sperm co-culture (3.5 and 35 ng/mL). Finally, the medium was removed and cells were trypsinized. Then, cells were washed twice with PBS^–/–^and resuspended in 300 μL PBS^–/–^. Cell viability was estimated using Trypan blue staining. The remaining cells were washed one more time by centrifugation at 300 *g* for 10 min at 4°C, lysed with Trizol (Invitrogen, Carlsbad, USA), and stored at -80°C until RNA extraction.

### Quantification of TGFB1 and IL10 in BOEC-conditioned media

The concentrations of TGFB1 and IL10 were assessed with the commercially available ELISA kits: bovine transforming growth factor β1 (CSB-E14208B) and bovine Interleukin 10 (CSB-E12917B) (Cusabio Biotech Co., LTD, Wuhan, China). A 100 μl of each medium sample was analyzed according to the manufacturer's instructions. To activate latent TGFB1 to the immune-reactive form, the medium was incubated with 1N HCL for 10 min at room temperature and the acidified samples were then neutralized by adding 13 μL of 1.2 N NaOH/0.5 M HEPES. All samples were run in duplicate. Optical density readings were performed at 450 nm.

### Quantification of PGE_2_ concentration

PGE_2_ concentrations were measured in the supernatant of BOECs after 24 h incubation and in the supernatant of PMN cultured for 4 h incubation of PMNs with BOEC-conditioned media, by using a competitive enzyme immune assay (EIA), as previously described [23]. Briefly, a 96-well ELISA plate (Corning, NY, USA) was coated with 100 μg of anti-rabbit IgG (Seikagaku, Tokyo, Japan) which was achieved after an incubation with polyclonal antibody solution (100 μl; 1:600,000) for 24 h at 4°C. The following day, plates were decanted and 15 μL of standards or samples were incubated with PGE_2_-HRP (1:40,000) overnight at 4°C. The coefficient of variance within and between assays was 7.3 and 11.4%, respectively. The ED50 values were 260 pg/mL and the ranges of the standard curves for these assays were 20–2000 pg/mL.

### Effect of BOEC-conditioned media (with and without sperm co-culture) in major pro- and anti-inflammatory cytokines expression of PMNs

PMNs were cultured in a 12-well plate at a density of 5 × 10^6^ cells/mL with BOEC-conditioned media (with and without sperm co-culture) for 4 h in a humidified atmosphere at 38.5°C in 5% CO_2_ (Fig 1). PMNs cultured in fresh media (DMEM/F12 with 0.1% FCS) were used as a control group. After the 4-h incubation, the supernatant was removed and the cells were collected and lysed using Trizol (Invitrogen, Carlsbad, USA) to then be stored at -80°C until RNA extraction.

### Effect of exogenous PGE_2_ in the immune-related gene expression of PMNs

PMNs were cultured in a 12-well plate at a density of 5 × 10^6^ cells/mL and incubated for 4 h in a humidified atmosphere at 38.5°C in 5% CO_2_ with different concentrations of PGE_2_ (3.5 and 35 ng/mL that are equivalent to 10^−8^ and 10^−7^ M). The cells were collected and lysed using Trizol (Invitrogen, Carlsbad, USA) and stored at -80°C until RNA extraction (*n* = 4).

### RNA extraction and cDNA synthesis

Total RNA was extracted from BOECs and PMNs using the Trizol reagent (Invitrogen, Carlsbad, USA) as previously described in the protocol of [27]. The RNA extracted from all samples was detected by ultraviolet (UV) spectroscopy (optical density, 260 nm) and the concentration was measured using a spectrophotometer (Eppendorf, Munich, Germany) at 260 and 280 nm absorbance values. The total extracted RNA was stored in RNA storage solution (Ambion, Austin, TX, USA) at –80°C until cDNA production. A DNase treatment step was performed using RQ1 RNase-Free DNase kit (Promega, Madison, WI, USA) to remove residual genomic DNA and other contaminations. The extracted RNA (1 μg) was incubated for 30 min at 37°C with 1 unit of the 10× RQ1 RNase-free DNase reaction buffer and 2 μL of the 1 μg/μL RNase-free DNase. To terminate the reaction, 1 μL of the RQ1 DNase Stop solution (20 mM EDTA) was added to the sample, and the mixture was incubated for 10 min at 65°C. First-strand cDNA synthesis was conducted according to the commercial protocol described in the SuperScript II Reverse Transcriptase kit (Invitrogen, Carlsbad, CA, USA). The mixture was prepared using 2 μL of the total RNA extracted from the sample (BOECs or PMNs), 1.5 μL of 50 ng/μL random primer (Invitrogen, Carlsbad, CA, USA), 1.5 μL of 10 mM PCR Nucleotide Mix (dNTP; Roche Diagnostics, Indianapolis, IN, USA), and 12 μL of H_2_O to obtain a total volume of 18 μL per sample. This mixture was then incubated at 65°C for 5 min in a thermal cycler (Bio-Rad, Munich, Germany). The samples were kept on ice while the second mixture, which consisted of 3 μL of 0.1 M dithiothreitol (DTT, Invitrogen, Carlsbad, CA, USA), 1.5 μL of 40 units/μL RNasin Ribonuclease Inhibitor (Promega, Madison, WI, USA), and 6 μL of 5× First-Strand Buffer (Invitrogen, Carlsbad, CA, USA), was added to each tube. The samples were incubated for 2 min at 42°C, and 0.2 μL of 200 units/μL SuperScript II Reverse Transcriptase was added to each tube. The thermal cycler was programmed at 25°C for 10 min, 42°C for 50 min, and then 70°C for 15 min. The synthesized cDNA was stored at -30°C.

### Real-time polymerase chain reaction (real-time PCR)

To quantify the mRNA expression of target genes (*IL10*, *TGFB1*, *TNF*, *IL1B*, *PTGES*, and *β-actin*), we used the synthesized cDNA to perform real-time PCR using a QuantiTect SYBR Green PCR Master Mix (QIAGEN GmbH, Hilden, Germany) by an iCycler iQ (Bio-Rad Laboratories, Tokyo, Japan). The amplification program was performed with an initial activation step (15 min at 95°C), followed by 40 cycles of PCR (15 sec denaturation at 95°C, 30 sec annealing at 55–58°C, and 20 sec extension at 72°C). The calculated cycle threshold (Ct) values were exported to Microsoft Excel to be analysed. Ct values were normalized using *β-actin* as the internal standard using the Delta-Delta comparative threshold method [28] to quantify the fold change between the samples. The expression of *β-actin* was stable in all experiments and cross treatments; no significant variations in *β-actin* expression were detected. Specific primers for each gene were designed using PRIMER EXPRESS software (Perkin-Elmer, Boston, MA) as shown in Table 1.

**Table 1:** Primers used to amplify specific bovine transcripts.

Gene		Sequence of nucleotide (5'⇒3')	Accession No.
***ß actin***	*F	TCACCAACTGGGACGACATG	AY141970.1
	*R	CGTTGTAGAAGGTGTGGTGCC	
***IL10***	F	GAGATGCGAGCACCCTGTCT	NM_174088.1
	R	GGCTGGTTGGCAAGTGGATA	
***TGFB1***	F	CTTTCTTCAAATGCAGCATTGG	NM_001166068.1
	R	GGGTCTGGGTGATACAACGAA	
***TNF***	F	CAAAAGCATGATCCGGGATG	NM_173966.3
	R	TTCTCGGAGAGCACCTCCTC	
***IL1B***	F	AATCGAAGAAAGGCCCGTCT	NM_174093.1
	R	ATATCCTGGCCACCTCGAAA	
***PTGES***	F	AAAATGTACGTGGTGGCCGT	NM_174443.2
	R	CTTCTTCCGCAGCCTCACTT	

* F, forward; R, reverse

### Immunohistochemistry

Only healthy oviducts were transported from the local slaughterhouse to the laboratory (immersed in 0.9% saline solution and placed in an icebox). The phase of the estrous cycle was identified as previously described by [23]. The oviducts were used from both the pre-ovulatory and post-ovulatory phases. Paraffin-embedded tissue sections (4 μm thick) of the bovine ampulla or isthmus were deparaffinised in xylene and rehydrated in descending concentrations of graded alcohols. The slides were rinsed in PBS (pH 7.2) and antigen retrieval was performed by incubating the section in an EDTA solution (10 mM Tris Base, 1 mM EDTA, 0.05% Tween 20, pH 9.0; 10 min at 96°C). After washing in PBS, slides were incubated in 20% normal goat serum (diluted in PBS) at room temperature for 20 min. Then, the slides were incubated overnight at 4°C with a TGFB1 antibody (rabbit anti-human TGFB1 antiserum; 1:75; sc146; Santa Cruz Biotechnology, USA) or IL10 antibody (rabbit anti-bovine IL10 antiserum; 1:100; PAA056Bo01; Uscn Life Science Inc., China) diluted in PBS containing 1% bovine serum albumin. For antigen detection, the EnVision System was used in accordance to the manufacturer’s protocol (DAKO, Glostrup, Denmark). Subsequently, sections were washed with PBS and the peroxidase activity was detected by incubation with a diaminobenzidine solution (DAB, Sigma-Aldrich, Steinheim, Germany) for 5 min at room temperature. Sections were counterstained with hemalum, dehydrated, and mounted with DPX Mountant (distrene, plasticizer, xylene, Fluka, Buchs, Switzerland). To analyse nonspecific binding, the primary antibody was replaced with rabbit IgG (Sigma-Aldrich, Steinheim, Germany) at the same concentration of the primary antibody (negative control).

### Statistical analyses

Data resulting from the time-dependent effect that the sperm-BOEC binding has on the relative mRNA expression of BOECs and TGFB1 production were analyzed and compared to the control values for each time point using a Two-Way ANOVA followed by the Bonferroni’s post hoc test. Statistical differences in all other data were determined using a Student’s *t*-test (for two groups; EIA for PGE_2_ on the BOEC-conditioned media) or One-Way ANOVA followed by Bonferroni’s post hoc test (for more than two groups; Effect of exogenous PGE_2_ in the immune-related gene expression of BOECs and PMNs, EIA data for PGE_2_ in the supernatant of cultured PMN, and the effect of BOEC-conditioned media on the cytokine expression of PMNs). All results were considered to be statistically significant at *P* < 0.05.

## Results

### Immunohistochemical localisation of TGFB1 and IL10 in bovine oviduct

**Fig 2:**
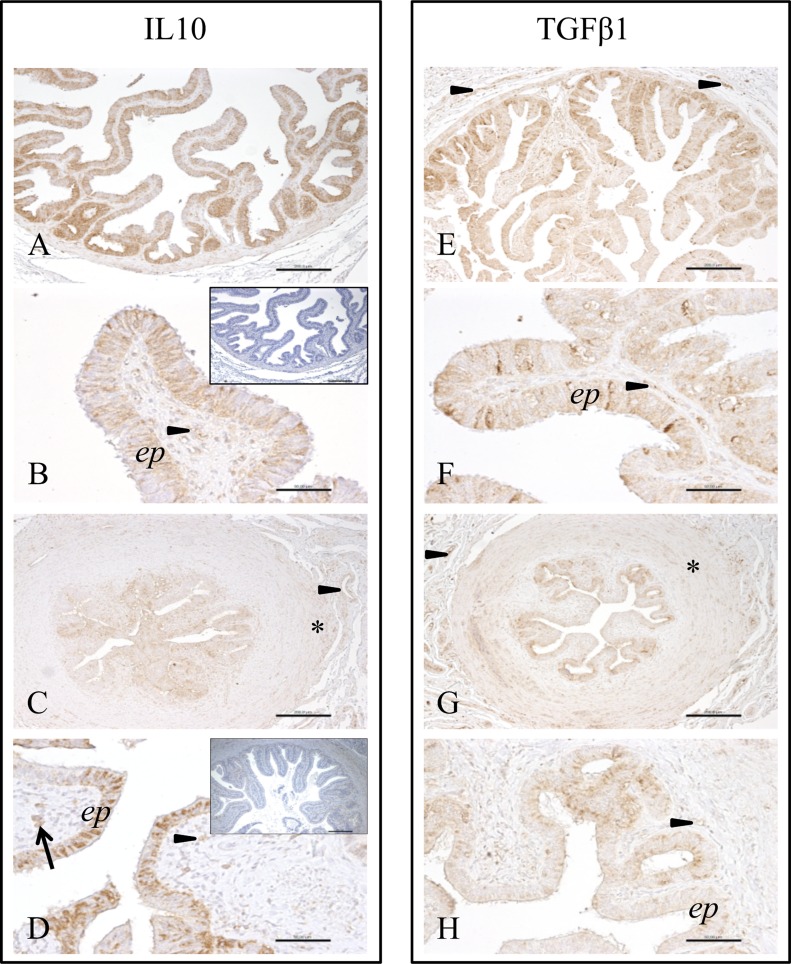
Immunohistochemical localization of IL10 and TGFB1 in bovine oviduct. Representative photos for immunohistochemical labelling of IL10 (A-D) and TGFB1 (E-H) in paraffin wax sections of the ampulla (A, B, E, F) and isthmus (C, D, G, H) of the bovine oviduct. Low magnification images (A, C, E, G) show a clear co-localization of both cytokines in epithelial cells (brown stain of luminal cell layer) and vessel walls of large blood vessels (arrowheads), while other tissue components are negative. High magnification images (B, D, F, H) reveal a cytoplasmic epithelial immunoreaction (*ep*) and staining of stromal endothelial cells (arrowheads). Besides, in the isthmus both proteins are expressed by smooth muscle cells of the surrounding prominent muscle layer (asterisks in C and G). Occasionally occurring leukocytes are positive for both cytokines (arrow in D). Insets to B and D show representative negative control sections. Scale bars represent 200 μm in A, C, E, G and 50 μm in B, D, F, H.

Immunohistochemistry was performed using oviduct tissue from pre-ovulatory and post-ovulatory phases. No stage-dependent differences were observed in either TGFB1 or IL10 expression. Positive labelling for both cytokines was detected mainly in the epithelial cells of the oviduct. Co-localisation of both cytokines occurred also in endothelial cells, smooth muscle cells of large blood vessel walls as well as some scattered leukocytes. In the isthmus both proteins were expressed by the surrounding muscle layer. Overall, the bovine oviduct evidently expressed TGFB1 and IL10 in both, the ampulla and isthmus throughout the estrous cycle (Fig 2).

### Sperm-BOEC binding modulates the gene expression for *TGFB1*, *IL10*, *PTGES*, *TNF* and *IL1B* in BOECs

**Fig 3:**
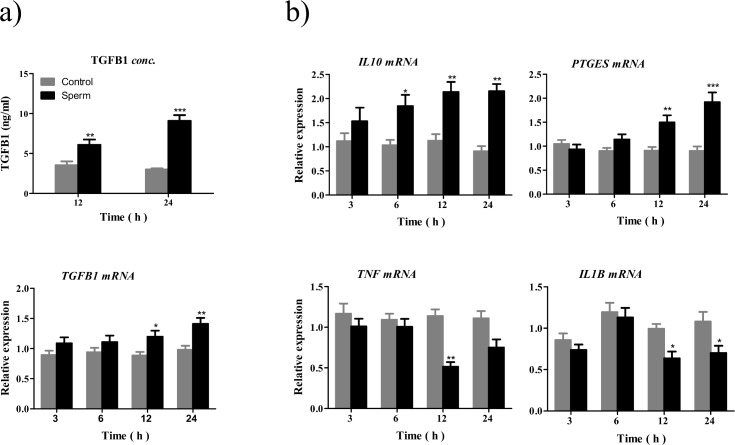
Effect of sperm-BOEC binding on TGFB1 production and the expression of anti- and pro-inflammatory cytokines and *PTGES* in BOECs. Viable sperm were co-cultured with BOEC monolayer for 3, 6, 12, or 24 h to detect the effect of sperm-BOEC binding. a) TGFB1 concentration in BOEC-conditioned media at 12 and 24 h by ELISA and its mRNA expression at different time points. b) mRNA expression of *IL10*, pro-inflammatory cytokines (*TNF* and *IL1B*), and *PTGES* in BOECs, as measured by real-time PCR. Results are presented as the mean ± SEM of five independent experiments performed in triplicate. All mRNA expression levels were normalized to *β-actin*. Asterisks denote statistical differences: * *P* < 0.05, ** *P* < 0.01, *** *P* < 0.001, when compared to the control at the same time point and determined using a Two-Way ANOVA followed by the Bonferroni's post hoc test.

BOECs expression showed no immediate changes in the cytokine mRNA expressions of our focused cytokines until 6 h from sperm-BOEC binding. There was a significant up-regulation of *TGFB1* at 12 and 24 h (*P* < 0.05) and of *IL10* at 6, 12, and 24 h (*P* < 0.05), while there was a down-regulation of *TNF* at 12 h (*P* < 0.001) and of *IL1B* at 12 and 24 h (*P* < 0.05). Additionally, *PTGES* gene expression was up-regulated at 12 and 24 h (*P* < 0.01) compared to that in the control (Fig 3).

### The increase in TGFB1 concentration in BOEC media after sperm co-culture

The concentrations of TGFB1 in BOEC-conditioned media that were collected after sperm co-culture at 12 and 24 h were significantly higher (P < 0.01 and P < 0.001, respectively) than those in controls (Fig 3A). In all media, IL10 concentration was below the detection limits of the ELISA assay (<5 pg/mL).

### Sperm-BOEC binding induces PGE_2_ release from BOECs

**Fig 4:**
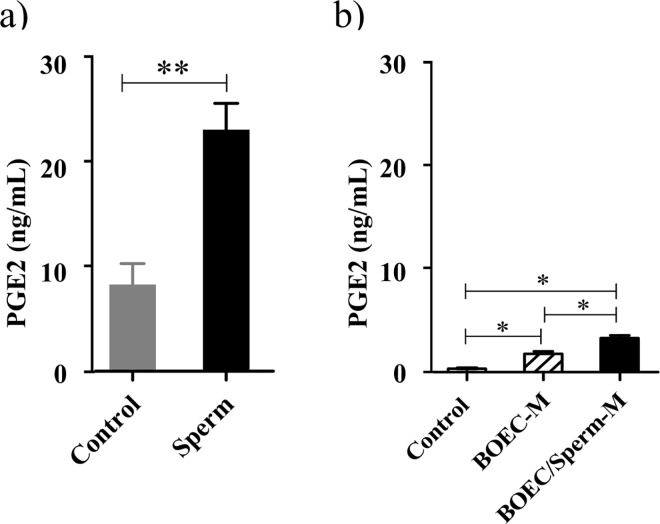
PGE_2_ secretion by BOECs and PMNs. a) PGE_2_ release by BOEC monolayers after 24 h of co-culture with and without sperm (n = 5). Data are presented as the mean ± SEM. Asterisks denote statistical differences: ** *P* <0.01, determined by a Student’s *t*-test. b) PGE_2_ release (mean ± SEM) by PMNs (5×10^6^ cells/mL) after 4 h incubation with BOEC-conditioned media (with and without sperm co-culture) (*n* = 8). The absolute production of PGE_2_ by PMNs was calculated after subtracting out the amount of PGE_2_ that was present in the BOEC-conditioned media. Asterisks denote statistical differences: * *P* < 0.05, as determined using a One-Way ANOVA followed by the Bonferroni’s post hoc test.

The binding of sperm to BOECs increased the release of PGE_2_ after 24 h (approximately three fold increase), when compared to control (*P* < 0.01, Fig 4A).

### Exogenous PGE_2_ down-regulates mRNA expression for *TNF*, *IL1B* in the BOECs

**Fig 5:**
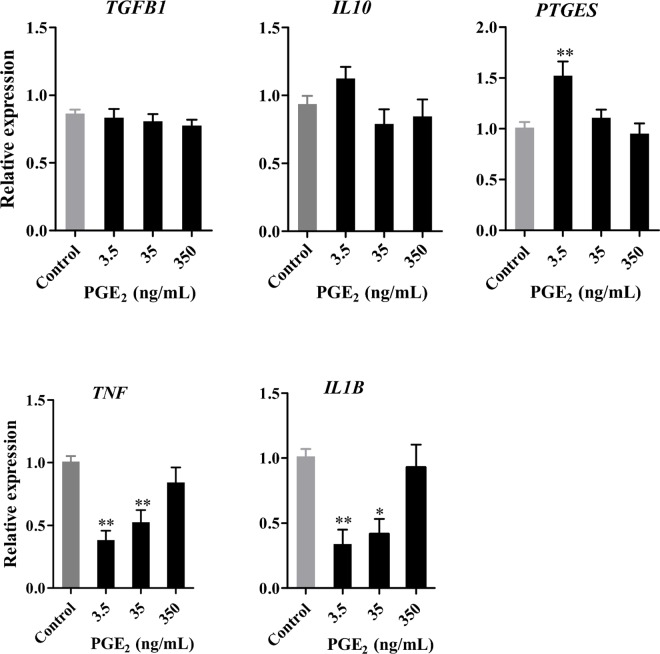
Effect of exogenous PGE_2_ on the expression of pro- and anti-inflammatory cytokines in BOECs. BOEC monolayers were exposed to different concentrations of PGE_2_ (3.5, 35 and 350 ng/mL) for 24 h. Results are presented as the mean ± SEM of five independent experiments performed in triplicates. All expression levels were normalized to *β-actin*. Asterisks denote statistical differences: * *P* < 0.05, ** *P* < 0.01, when compared to the control and determined using a One-Way ANOVA followed by the Bonferroni's post hoc test.

Addition of PGE_2_ to BOECs suppressed the expression of *TNF* and *IL1B* (pro-inflammatory cytokines) at concentrations of 3.5 and 35 ng/mL (P < 0.05, Fig 5), but not at 350 ng/mL. PGE_2_ did not affect the expression of anti-inflammatory cytokines such as TGFB1 and IL10. Notably, the *PTGES* expression was up-regulated only at the lowest concentration of PGE_2_ (3.5 ng/mL; P < 0.01, Fig 5).

### BOEC-conditioned media suppress the mRNA expression of *TNF* and upregulate *TGFB1* and *IL10* expressions in PMNs

**Fig 6:**
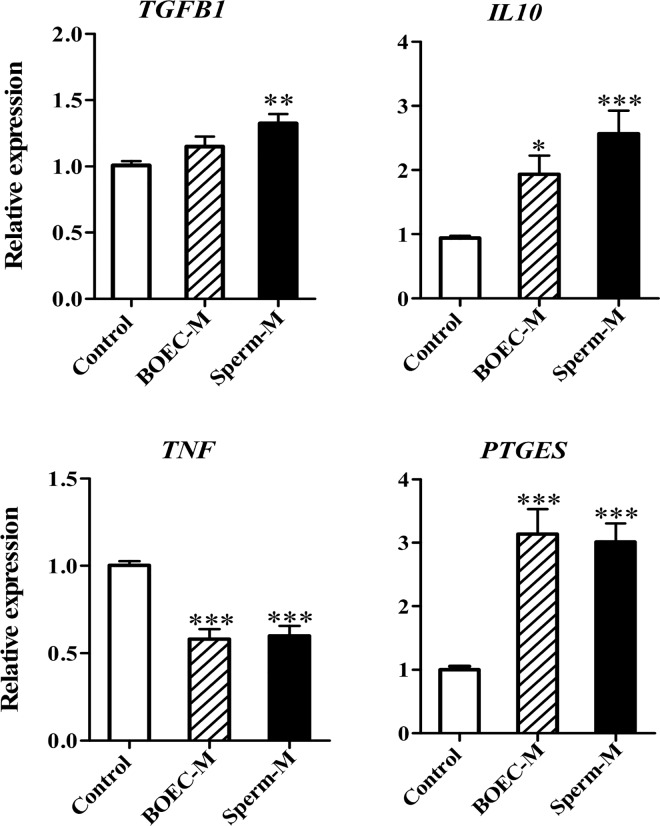
Changing of PMNs gene expression after culturing in BOEC-conditioned media. Cytokine gene expression (*TGFB1*, *IL10*, *TNF*, and *PTGES*) in PMNs incubated for 4 h with BOEC-conditioned media (with and without sperm co-culture). Results are presented as the mean ± SEM of three independent experiments performed in duplicate. All expression levels were normalized to *β actin*. Asterisks denote statistical differences: * *P* < 0.05, ** *P* < 0.01, *** *P* < 0.001, when compared to the control, as determined using a One-Way ANOVA followed by Bonferroni's post hoc test.

BOEC-conditioned media (with and without sperm co-culture) suppressed the expression of *TNF* (*P* < 0.001) in PMNs. The medium obtained from BOECs co-cultured with sperm stimulated the expression of the *TGFB1* (anti-inflammatory cytokines; *P* < 0.01) and *IL10* (*P* < 0.001). Moreover, *PTGES* gene expression was significantly up-regulated using both media (*P* < 0.001, Fig 6).

### BOEC-conditioned media induce PGE_2_ release from PMNs

The overall PGE_2_ production by PMNs significantly increased after 4 h incubation with BOEC-conditioned media, when compared to fresh media (*P* < 0.001). Moreover, BOEC-conditioned media co-cultured with sperm induced additional PGE_2_ production by PMNs (P < 0.001, Fig 4B).

### Exogenous PGE_2_ induces similar effect of BOEC-conditioned media in the PMNs gene expression

**Fig 7:**
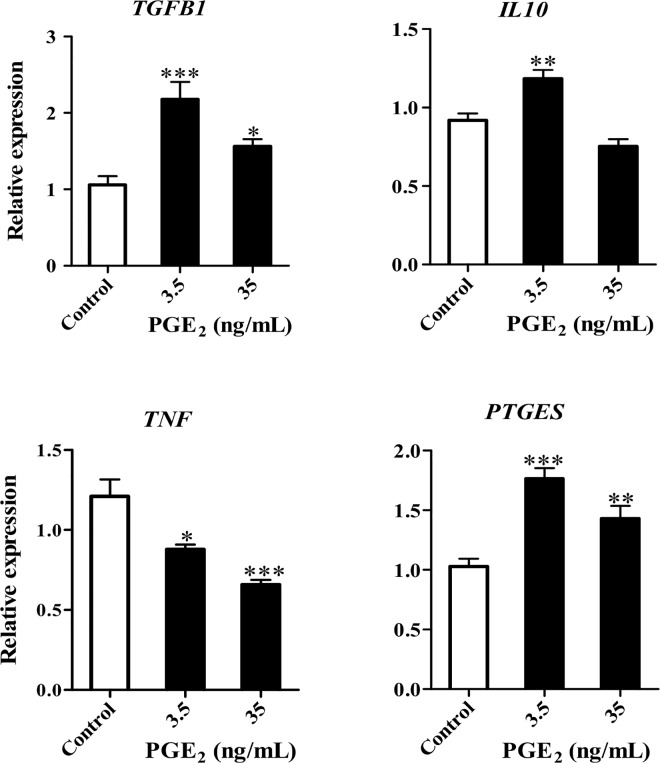
Effects of exogenous PGE_2_ on the gene expression in PMNs. Pro- and anti-inflammatory cytokines and *PTGES* in PMNs cultured for 4 h with different concentrations of PGE_2_. Results are presented as the mean ± SEM of four independent experiments performed in triplicate. All expression levels were normalized to *β actin*. Asterisks denote statistical differences; * *P* < 0.05, ** *P* < 0.01, *** *P* < 0.001, when compared to the control, as determined using a One-Way ANOVA followed by Bonferroni’s post hoc test.

PGE_2_ at concentrations of 3.5 and 35 ng/mL significantly suppressed *TNF* expression and induced the expression of *TGFB1* and *PTGES* (P < 0.05, Fig 7). At 3.5 ng/mL, PGE_2_ stimulated *IL10* expression (P < 0.05, Fig 7).

## Discussion

For fertilization to take place, it needs all the essential requisites in the reproductive system as well as the immune system. The present *in vitro* data may illustrate the oviduct’s anti-inflammatory response to the sperm and the crucial role of PGE_2_ in modulating the local innate immunity of the bovine oviduct, by its actions on both epithelial cells and PMNs under physiological conditions, even if the study used simplified cell culture model.

Immunohistochemistry results showed that TGFB1 and IL10 (anti-inflammatory cytokines) are strongly expressed in the bovine oviduct epithelial and also endothelial cells of the surrounding vasculature (in both isthmus and ampulla) throughout the estrous cycle. The same localization was described for both cytokines in the gilt oviduct [29]. Blois et al. [30] proposed that IL10 and TGFB1 activate the production of pregnancy-protective Th2/Th3 cytokines which enhance suppression of the immune system, thereby contributing to fetal tolerance. This idea is supported by the basic findings of Pomié *et al*. [31] and Kapp and Bucy [32] who reported that CD8+ regulatory T lymphocytes suppress the immune reactivity by production of IL10 and/or TGFB in mice and human. The present results indicate that TGFB1 and IL10 might have a crucial role in the bovine oviduct for maintenance of tolerance to non-self cells (sperm and embryos) and for regulation of immunological homeostasis [12].

In cows, it takes about 6–8 h until enough numbers of sperm reach the oviduct for successful fertilization [33]. Sperm attach to the apical plasma membrane of the ciliated and secretory epithelial cells and remain as a reservoir where this attachment mediated by multiple factors [2,34]. This reservation time is essential to ensure enough number of fertile sperm in the oviduct when ovulation occurs. Wilmut and Hunter [35] showed that the population of sperm capable of fertilization is established in the bovine oviduct not less than 6 h after artificial insemination but probably more than 12 h, and those sperm can remain arrested in the isthmus for ≥18 h and only detach from the epithelium near the time of ovulation. Moreover, Fazeli *et al*. [17] revealed that a changing of the oviductal transcriptome profiles at arrival of sperm was shown in mice. In pigs, sperm can change gene expression profiles and regulate secretory proteins in the oviduct *in vivo* [18]. Therefore, in the present study, BOECs were collected at different time points (3, 6, 12, and 24 h) after sperm-BOEC binding. The data revealed that no immediate changes occurred in the mRNA expression of our focused immune-related cytokines in BOECs. However, at 6, 12, and 24 h, there was a significant up-regulation of *TGFB1* and *IL10* (anti-inflammatory cytokines), while there was down-regulation of *TNF* and *IL1B* (pro-inflammatory cytokines), especially after 12 h. These findings suggest a possible cross-talk between sperm and BOECs, through the induction of such anti-inflammatory cytokines, to tolerate sperm *in vivo* while remaining intolerant to pathogens [18].

In accordance with its mRNA expression, TGFB1 protein level was increased after sperm-BOEC binding at 12 and 24 h time points (P < 0.01 and P < 0.001, respectively). Jiwakanon *et al*. [29] supposed that the significant presence of TGFB1 in the porcine oviduct may induce a down-regulation of the immune response for sperm protection.

Sperm-BOEC binding induced *PTGES* expression in a time-dependent manner, which enhanced the secretion of PGE_2_ from the BOECs, consistent with previous results from our laboratory [22]. Moreover, PGE_2_ at the physiological concentrations (3.5 and 35 ng/mL) found in the pre-ovulatory stage [10] strongly suppressed the expression of *TNF* and *IL1B* (pro-inflammatory cytokines) in BOECs in culture without any effects on the expression of *TGFB1* and *IL10* (anti-inflammatory cytokines). In T-cells, PGE_2_ favours anti-inflammatory (Th2) cytokine profiles by inhibiting the production of the inflammatory (Th1) cytokines [36]. The synergism between PGE_2_ and TGFB1 was reported for suppression of immune responses and maintaining tolerance [37]. Thus, PGE_2_ appears to drive the immunosuppressive state with TGFB1 in the bovine oviduct.

**Fig 8:**
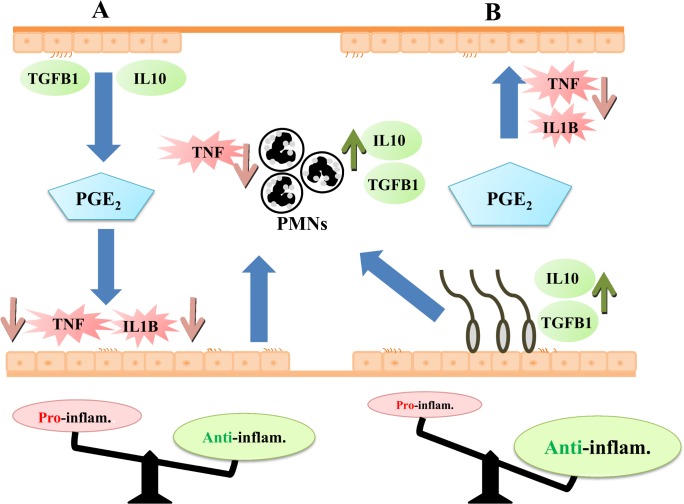
Schematic illustration to show the local oviductal immunity after sperm binding to BOECs. Based on the present results, sperm-BOEC binding could regulate the oviduct immunological environment under physiological conditions. (A) BOECs strongly express TGFB1 and IL10. Basal PGE_2_ levels in oviductal cells downregulate pro-inflammatory cytokines (TNF and IL1B). (B) Sperm-BOEC binding enhances the production of PGE_2_ and stimulates anti-inflammatory cytokines (TGFB1 and IL10), which strengthens the anti-inflammatory environment needed for sperm protection and survival. PGE_2_ secreted from BOECs decreases TNF expression in PMNs; furthermore, the sperm-BOEC binding induces further secretion of PGE_2_ in BOECs. PGE_2_ production results in the up-regulation of TGFB1 and IL10, which may contribute to supply a stable anti-inflammatory environment in the oviduct.

The presence of PMNs in the bovine oviduct flush (pre-ovulatory stage) points to the possibility of sperm phagocytosis, thereby reducing the quantity of viable sperm in the oviduct [10]. However, the present study showed that BOEC-conditioned media (either with or without sperm co-culture) induced *TGFB1* and *IL10* (anti-inflammatory cytokines) expressions in PMNs. In particular, the sperm-BOEC binding intensified this response; therefore, it is possible that the bovine oviduct basically suppresses local innate immunity, and the presence of sperm continues to strengthen this anti-inflammatory environment (Fig 8). Interestingly, we found that the BOEC-conditioned media stimulates the release of PGE_2_ from PMNs. Furthermore, the media after sperm-BOEC binding further stimulated PMNs to secrete PGE_2_. However, taking into consideration the relatively high number of PMNs used in the present culture model (5 × 10^6^ cells/mL), the contribution of PMNs in the secretion of PGE_2_ appears to be minimum. In addition, PGE_2_ at physiological levels in the bovine oviduct seems to suppress the expression of *TNF* and induce the expression of anti- *TGFB1* and *IL10* in PMNs.

It is interesting to note that only 3.5 ng/mL of PGE_2_ up-regulates *PTGES* expression in PMNs, suggesting that a physiological concentration of PGE_2_ is able to stimulate its production in oviductal PMNs, while higher doses of PGE_2_ may induce negative feedback loop, thus decreasing its production. Importantly, the effects of exogenous PGE_2_ on PMNs mimicked the effect of the BOEC-conditioned media with the sperm co-culture (stimulation of *TGFB1* and *IL10*, and suppression of *TNF*). PGE_2_ can suppress phagocytosis, nitric oxide production, and intracellular death of different immune cell populations [10,38,39]. Therefore, it is likely that PGE_2_ biosynthesis in BOECs regulate the immunological environment to support sperm survival in the bovine oviduct.

Altogether, our findings show the up-regulation of TGFB1 and IL10 expression with the increase in the secretion of TGFB1 and PGE2 in BOEC after sperm binding. PGE2 could modulate the expression of immune related cytokines in both BOEC and PMNs in the present in vitro model. Thus, the present results support the concept that sperm are able to create their own favourable environment in the oviduct; furthermore, they may protect themselves from immunological attack to keep a significant number of sperm for a successful fertilization. Further *in vivo* studies by using heifers after artificial insemination are required to confirm our notion.
